# Pediatric trichodysplasia spinulosa in skin of color

**DOI:** 10.1016/j.jdcr.2025.03.038

**Published:** 2025-05-29

**Authors:** Catherine Laferté, Jérôme Coulombe, Sélia Kearns-Turcotte, Yasmine Yesli, Anne-Laure Lapeyraque, Maryam Piram

**Affiliations:** a Division of Dermatology, Department of Medicine, CHUM, Université de Montréal, Montréal, Quebec, Canada; b Division of Dermatology, Department of Pediatrics, CHU Sainte-Justine, Université de Montréal, Montréal, Quebec, Canada; c Division of Dermatology, Department of Medicine, CHUL, Universite Laval, Quebec City, Quebec, Canada; d CHU Sainte-Justine Research Center, Université de Montréal, Montréal, Quebec, Canada; e Nephrology and Hemodialysis Service, Department of Pediatrics, CHU Sainte-Justine, Montréal, Quebec, Canada

**Keywords:** cidofovir cream, facial eruption, folliculocentric keratin spines, immunosuppression, isotretinoin, madarosis, nose dermatitis, pediatric dermatology, polyomavirus infection, skin of color, tacrolimus, trichodysplasia spinulosa, trichodysplasia spinulosa-associated polyomavirus, viral dermatosis

## Introduction

Trichodysplasia spinulosa (TS) is a rare, viral-induced condition observed in immunocompromised individuals, caused by the TS-associated polyomavirus (TSPyV). Clinically, TS typically presents as asymptomatic or occasionally pruritic follicular papules and spines, primarily involving the central face. Scattered hyperkeratotic papules may also appear on other areas of the body. Despite its distinct clinical features, TS remains underrepresented in skin of color (SOC) literature, with limited documentation on presentation and treatment outcomes in pediatric populations. This report aimed to address this gap by describing an uncommon pediatric case of TS in a child with SOC and highlighting key differences in clinical presentation, management, and outcomes.

## Case report

**Fig 1:**
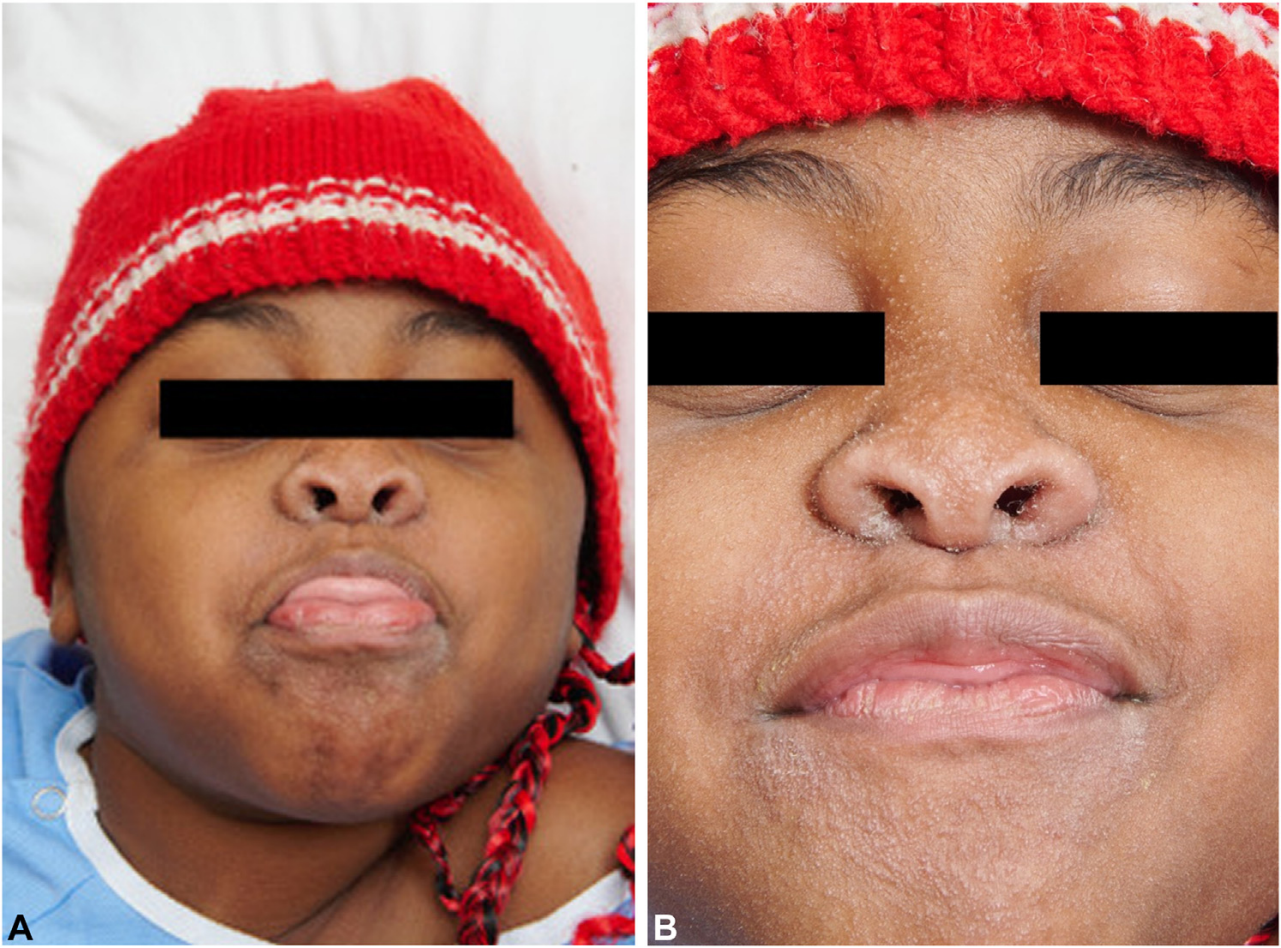
(**A, B**) Initial presentation of trichodysplasia spinulosa: The patient presented with an asymptomatic, infiltrative, monomorphic, flesh-colored micropapular eruption localized to the central face, with scattered involvement of the preauricular and chin areas.

**Fig 2:**
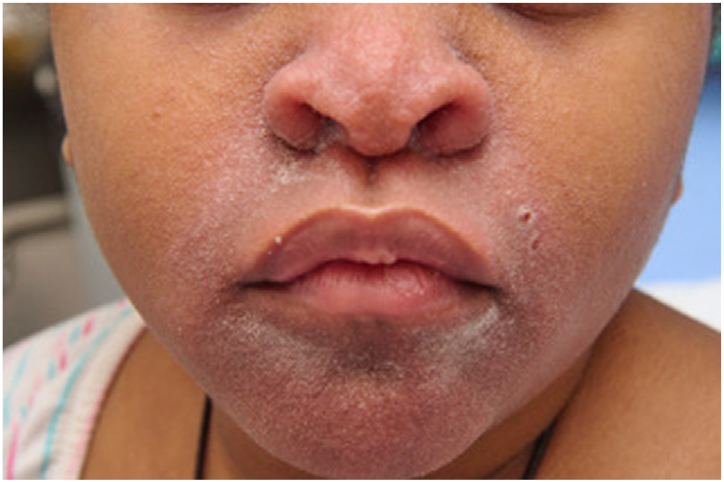
Progression of trichodysplasia spinulosa: Worsening folliculocentric keratin spines, madarosis, and significant nasal infiltration.

**Fig 3:**
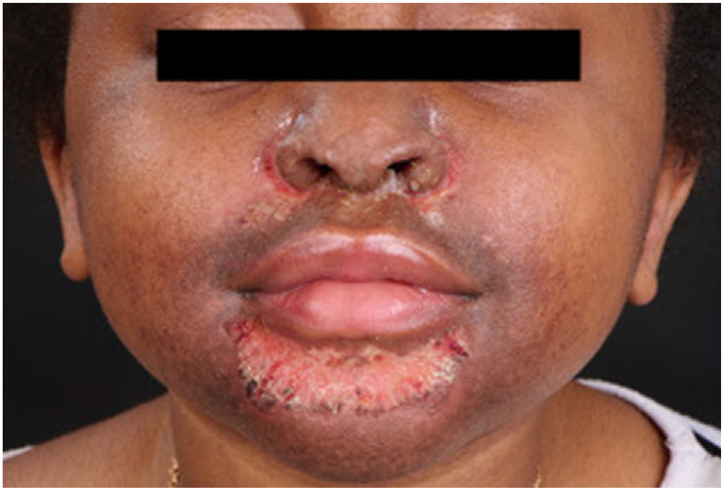
Transient erosions under cidofovir 1% cream.

**Fig 4:**
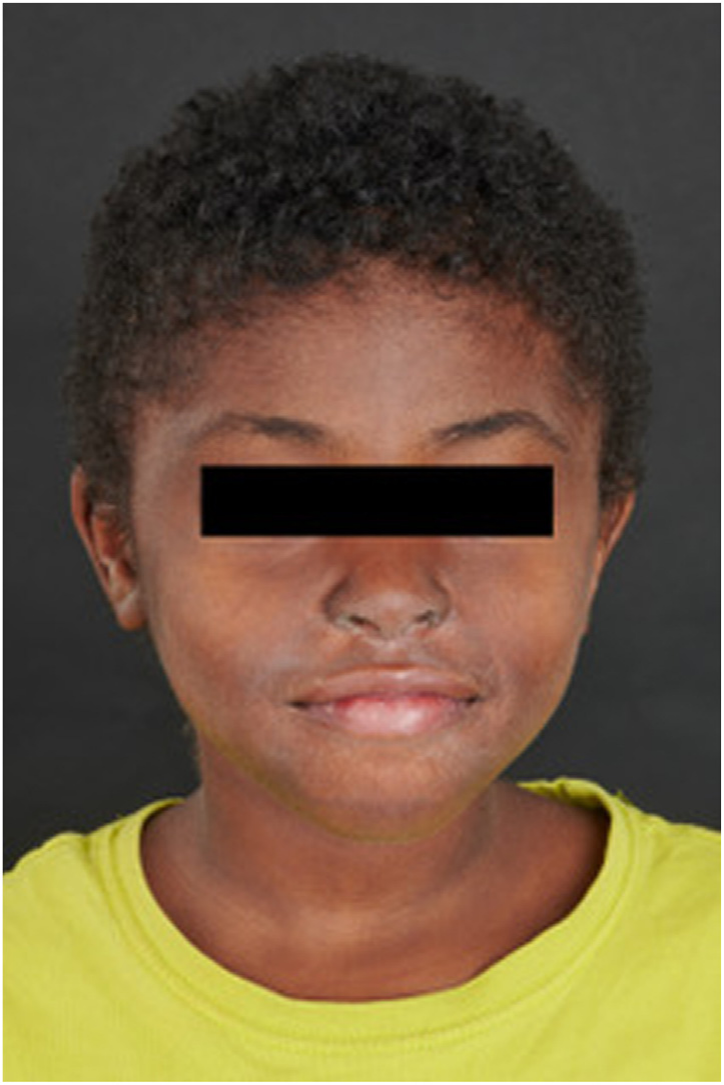
Resolution of trichodysplasia spinulosa: Significant improvement after treatment with twice-daily 1% topical cidofovir, with near-complete resolution of follicular spines and resolution of facial infiltration.

We present the case of an 8-year-old girl with SOC who developed a centrofacial eruption. Her medical history was significant for COPA syndrome, a monogenic autoinflammatory interferonopathy, complicated by anti neutrophil cytoplasmic antigen/myeloperoxydase-positive grade 4 glomerulonephritis requiring a renal transplant. Her ongoing systemic medications included tacrolimus, mycophenolate mofetil, prednisone, and granulocyte colony-stimulating factor. The patient presented with an asymptomatic, infiltrative, monomorphic, flesh-colored micropapulareruption localized to her central face, with scattered involvement of the preauricular and chin areas (Fig 1). She also had follicular papules on her shoulders and trunk resembling atopic follicular eczema. Initial empirical treatments with metronidazole cream, oral doxycycline, and oral isotretinoin failed. The eruption progressed to prominent folliculocentric keratin spines, madarosis, and disfiguring nasal infiltration (Fig 2). A 3-mm skin punch biopsy of the nose revealed dilatation and keratotic plugging of vellus hair infundibula, with dystrophic expansion of the inner root sheath and absence of hair shafts. Viral polymerase chain reaction studies on the biopsy tested positive for TSPyV. Topical cidofovir 1% cream was selected for treatment and was applied twice-daily to the central face until resolution. Significant improvement was observed within 4 weeks, despite transient nonscarring erosions during treatment (Fig 3). The inflammatory reaction to topical cidofovir did not result in scarring or permanent hypopigmentation (Fig 4). Therefore, even with marked skin inflammatory reaction to treatment, topical cidofovir achieved near-complete resolution of TS without permanent sequela in our patient with SOC. Cidofovir also cleared TS mimicking follicular atopic dermatitis on the trunk and shoulders of our patient.

## Discussion

TS is a viral-induced condition associated with drug-induced immunosuppression, predominantly reported in solid organ transplant recipients.^1^ It is caused by TSPyV, with its small invading T antigen believed to disrupt follicular keratinocyte cycling.^2^^,^^3^ TSPyV in contrast to the wider tropism of BK and JC polyomaviruses has a distinct affinity for the body’s pilosebaceous units, causing TS with its hallmark facial keratinous follicular spines and alopecia from altered hair shafts.^1^^,^^4^ Although TS predominantly affects the central face, variations in clinical presentation may exist based on skin phototype. In SOC, lesions may appear more translucent and shinier rather than erythematous, as observed in our patient.

The transmission and pathogenesis of TSPyV remain poorly understood, although its detection in nasopharyngeal and fecal samples suggests potential respiratory and/or fecal-oral transmission routes.^5^^,^^6^ Although polyomaviruses such as John Cunningham polyomavirus and BK polyomavirus are typically thought to emerge through reactivation in immunosuppressed individuals, evidence from TS suggests a different mechanism. Anti-TSPyV antibodies are only detectable after clinical signs of TS, hinting that TS may result from a primary infection in an immunosuppressed patient rather than reactivation.^6^ This hypothesis is further supported by the absence of viral-associated TS cases reported in the elderly, a population often affected by viral reactivations. For instance, despite a high adult seroprevalence of approximately 70% and the ubiquity of TSPyV similar to other polyomaviruses, only a few cases of TS have been reported globally.^5^ In the Netherlands, between 2010 and 2015, as little as 3 TS cases occurred compared with roughly 250 cases of BK polyomavirus-associated nephropathy during the same period.^2^^,^^7^ This discrepancy suggests that clinically overt TS may primarily arise from new infections in vulnerable hosts rather than reactivation of latent virus. This observation highlights the critical importance of equipping clinicians with the ability to recognize and manage this disease in a seronegative susceptible pediatric population.

TS, although nonlethal, reflects profound immunosuppression and can result in significant disfigurement warranting treatment. In our case, the scarcity of literature on pediatric TS, along with the patient's rare interferonopathy and kidney transplant, posed challenges in choosing a suitable therapy.^8^^,^^9^

This case highlights several uncommon aspects of TS in SOC, including distinct clinical features, challenges in diagnosis, and the successful use of twice-daily 1% topical cidofovir combined with tailored immunosuppression. The transition from mycophenolate mofetil to oral sirolimus, made by the patient’s infectious and nephrology teams, further underscores the importance of individualized immunosuppressive regimens to balance disease control and infection risk. Increased awareness of TS in SOC is crucial to improving recognition, diagnosis, and treatment outcomes in this underrepresented population. Increased awareness of TS in SOC is essential to improving diagnosis and treatment outcomes in this underrepresented population.

## Conflicts of interests

None disclosed.
